# Binary dose level classification of tumour microvascular response to radiotherapy using artificial intelligence analysis of optical coherence tomography images

**DOI:** 10.1038/s41598-022-18393-4

**Published:** 2022-08-17

**Authors:** Anamitra Majumdar, Nader Allam, W. Jeffrey Zabel, Valentin Demidov, Costel Flueraru, I. Alex Vitkin

**Affiliations:** 1 Department of Medical Biophysics, University of Toronto, Toronto, Canada; 2 Geisel School of Medicine, Dartmouth College, Lebanon, USA; 3 National Research Council Canada, Information Communication Technology, Ottawa, Canada; 4 Department of Radiation Oncology, University of Toronto, Toronto, Canada

**Keywords:** Microbiology, Physics, Applied physics, Biological physics, Electronics, photonics and device physics, Optical physics, Techniques and instrumentation, Optics and photonics, Applied optics, Lasers, LEDs and light sources, Optical physics, Optical techniques, Other photonics, Mathematics and computing, Computational science, Computer science, Software, Statistics, Engineering, Biomedical engineering, Medical research, Preclinical research, Computational biology and bioinformatics, Computational platforms and environments, Data acquisition, Data integration, Data processing, Databases, Image processing, Machine learning, Programming language, Software, Cancer, Cancer imaging, Cancer metabolism, Cancer microenvironment, Cancer models, Tumour heterogeneity, Biophysics, Biological fluorescence, Computational biophysics

## Abstract

The dominant consequence of irradiating biological systems is cellular damage, yet microvascular damage begins to assume an increasingly important role as the radiation dose levels increase. This is currently becoming more relevant in radiation medicine with its pivot towards higher-dose-per-fraction/fewer fractions treatment paradigm (e.g., stereotactic body radiotherapy (SBRT)). We have thus developed a 3D preclinical imaging platform based on speckle-variance optical coherence tomography (svOCT) for longitudinal monitoring of tumour microvascular radiation responses in vivo. Here we present an artificial intelligence (AI) approach to analyze the resultant microvascular data. In this initial study, we show that AI can successfully classify SBRT-relevant clinical radiation dose levels at multiple timepoints (t = 2–4 weeks) following irradiation (10 Gy and 30 Gy cohorts) based on induced changes in the detected microvascular networks. Practicality of the obtained results, challenges associated with modest number of animals, their successful mitigation via augmented data approaches, and advantages of using 3D deep learning methodologies, are discussed. Extension of this encouraging initial study to longitudinal AI-based time-series analysis for treatment outcome predictions at finer dose level gradations is envisioned.

## Introduction

Radiation therapy (RT) is a common option for the treatment of cancer^1^. Usually, a low dose is administered at regular intervals for better efficiency and normal tissue sparing as compared to a single high dose^2^. Recently, stereotactic body radiotherapy (SBRT) has been developed that utilizes an increased dose per fraction over fewer fractions, with significant clinical potential and economic advantages^3^. As doses increase to the levels commonly used in SBRT, the “conventional” radiobiology of tumour damage through cellular DNA breaks is insufficient to explain the observed effects, and additional mechanisms of action have been suggested^4–7^. Specifically, microvascular damage appears to assume an increasingly larger role at doses > 8–10 Gy. However, there is a complex and poorly understood interplay between the dose levels and temporal trajectory of the resultant microvascular changes.

Our lab has thus developed an in-vivo contrast-agent-free 3D microvascular imaging platform using speckle variance optical coherence tomography (svOCT)^8^, suitable for detailed preclinical RT studies of these effects^9^, with potential clinical implications^10,16^. The quantification of the resultant 3D microvascular images remains challenging however, as the derivation/selection of suitable biomarker(s) of the visualized blood microcirculation network is not obvious. A common approach is to derive analytical metrics such as vascular volume density, fractal dimension, vessel diameter proportion, average inter-vessel spacing and so on^11,12^.

While useful in some scenarios^9,11–16^, analytical biomarker derivation is challenging because (1) a significant amount of image post-processing is required, (2) often user-dependent error-prone steps such as skeletonization and binarization are involved, (3) choice of metric(s) is not obvious and may require clinical expertise, and (4) the selected metrics are unlikely to capture or reflect the full 3D heterogeneous complexity of the tumour microvascular network as they are often summarized by a single number representing the entire imaged volume. In this study, we chose to explore artificial intelligence (AI) approaches to microvascular image quantification which could potentially circumvent these analytical challenges.

Towards this goal, machine learning and deep learning techniques can be applied. Convolution neural networks (CNNs) have become the gold standard for image classification in recent years due to their sensitivity to fine details in images and are best suited for working with 3D data. These have the potential to capture the intricate information contained in the rich 3D svOCT volumes with minimal manual intervention. A CNN learns this information in the form of features automatically by training its nodes with different weights^17^ so that it can in turn predict outcomes such as tumour progression and/or other clinical treatment results systematically. However, CNNs require large data sets to ensure proper implementation. Applications of artificial intelligence in OCT are most common in ophthalmology such as the detection of age-related macular degeneration^18–20^, anomaly identification in retinal images^21^, prediction of visual acuity in patients with retinal disease^22,23^, automatic segmentation of corneal endothelial cells^24^, and capturing the vascular flow in retinal imaging^25^. Breast cancer is also one of the more popular application areas with multiple OCT-AI implementations for detection, assessment, and analysis^26–31^. Although rarer, research has been done in the context of brain^32,33^, colon^34,35^, skin^36^, and oral cancers^37^ as well. These initial results suggest that the microstructural and microvascular content of speckled OCT images may be successfully analyzed by AI approaches, primarily in the context of 2D B-scans and maximum intensity projections^18,19,21–25,27,28,30–37^, but also when considering full volumetric 3D scans ^20,26,29^.

To demonstrate the utility of AI in the context of our “shedding light on radiotherapy” OCT microangiography research, we present the results of a supervised binary classification study. The goal pursued here was to distinguish between the svOCT images of the tumour vasculature according to either low (10 Gy) or high (30 Gy) radiation dosages at multiple time points ranging from t = 2–4 weeks after irradiation. A CNN model, using the full 3D image volumes of the tumour vasculature as the input, was chosen for this task. The encouraging results of this pilot investigation suggest that AI may be useful in the context of svOCT-based quantification of microvascular radiobiology, and the developed analysis pipeline will be further adapted to address more challenging and interesting questions dealing with radiotherapy longitudinal response monitoring and outcome prognostication.

## Methodology

All animal procedures were performed in accordance with appropriate guidelines and regulations under protocol approved by the University Health Network Institutional Animal Care and Use Committee in Toronto, Canada (AUP #3256). The methods in this study are reported in compliance with the ARRIVE guidelines.

The data employed in this study consisted of OCT-imaged fluorescently-labelled patient-derived xenograft cell-line of pancreatic cancer (BxPC3) grown in mice dorsal skinfold window chambers (DSWC) having undergone high single-dose irradiation ranging between 10 and 30 Gy^9^. All OCT acquisitions were performed on a previously described custom-built swept-source system employing a Mach–Zehnder interferometer with quadrature phase detection. Using a Gaussian beam source centered at 1320 nm with 110 nm bandwidth, the OCT achieves a resolution of ~ 8 μm axially (in air) and ~ 15 μm laterally up to ~ 2.03 mm in depth. With an A-scan acquisition rate of 20 kHz and 80% lateral scanning galvanometer duty cycle, a B-scan rate of 40 frames per second is reached. At an inter-frame time of 25 ms, this enables rapid repeated scans per lateral step of the galvanometer required for svOCT processing^8^. Acquisitions with 6 × 6 mm^2^ FOV with 8 repeated B-scans per location generated volumetric scans approximately 800 × 1600 × 8 × 500 voxels (lateral × lateral × B-scan repetitions × depth) in approximately 11 minutes, primarily limited by the write speed of the DAQ computer (6 core i7-6700K CPU running at 3.40 GHz with 64 GB RAM). Lateral oversampling is performed to mitigate potential motion artifacts along the slow scanning direction of the galvanometer. Post-acquisition, the temporal variance is computed at every spatial voxel according to the speckle variance algorithm to enhance vascular contrast and thus visualize tissue microvasculature^8^; subsequently, averaging is performed laterally yielding 800 × 800 × 500 voxels in approximately 5 minutes. Following application of the binary volumetric tumour mask to remove the glass and air gap over the tissue surface, as well as the surrounding non-tumour tissue, the data is resized to 200 × 200 × 400 voxels as the required input data size for the CNN.

For this deep learning analysis, the raw OCT 3D data scans only underwent speckle variance processing to enhance vascular contrast followed by volume of interest (VOI) selection, fluid buildup removal, and denoising^38^ (see below). The post-processing pipeline used to carry out these tasks has been previously created in MATLAB (MathWorks, Natick, MA). The CNN classifier implementations and subsequent analyses have been developed in Python 3.7 using open-source libraries.

Before using the 3D images for the CNN model, it was necessary to limit the analysis only to the imaged field of view that actually contains irradiated tumour vessels. VOIs were therefore selected that encompassed primarily the tumour vasculature, excluded the surrounding healthy tissue, and the air gap between the glass and tumour surfaces. This is important so as not to have a vast feature space with noncontributory information, and the CNN model unable to learn the relevant discriminatory information as a result. Consequently, confining the analysis to tumour-tissue-specific VOIs was done to ensure proper performance of the models.

**Figure 1:**
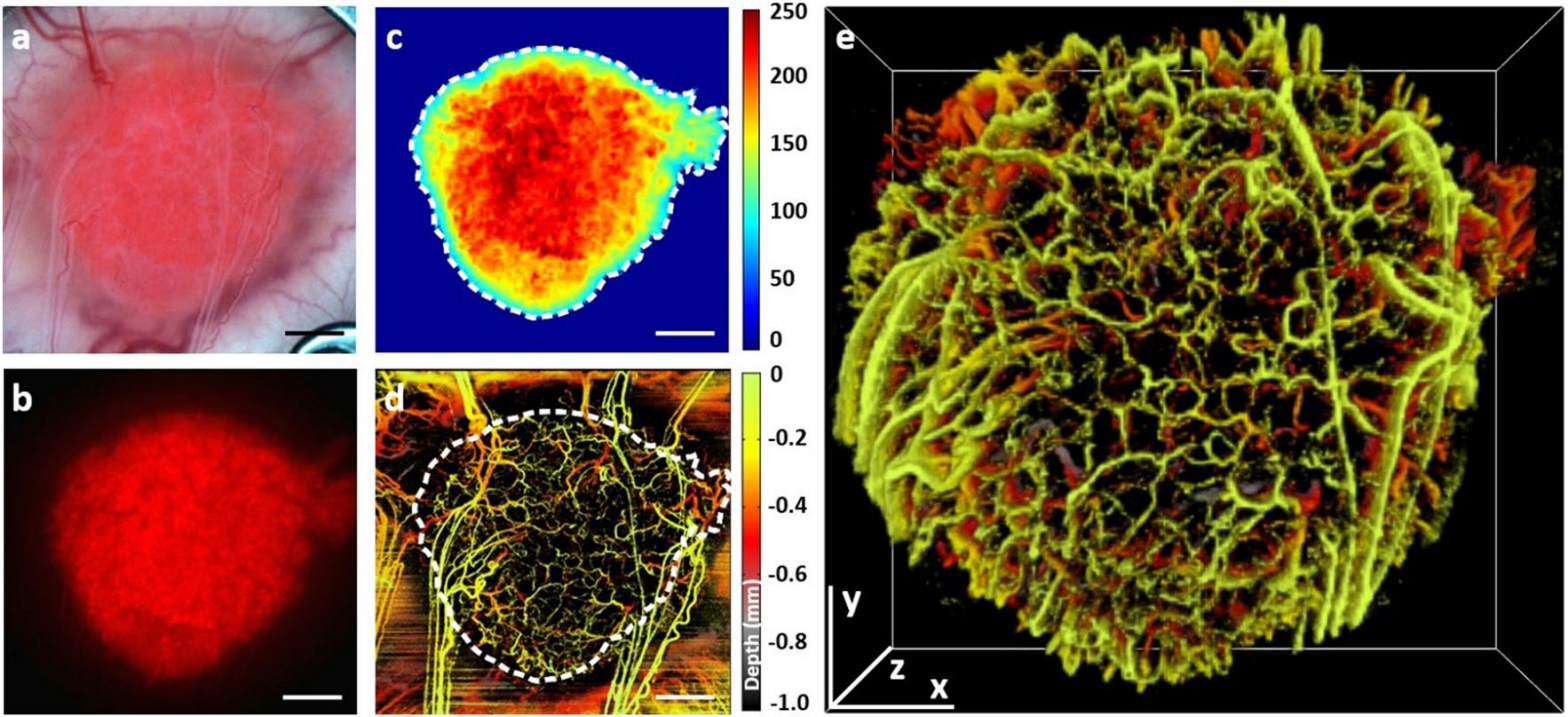
Tumour volume of interest (VOI) selection for the convolution neural network classifier implementation: (**a**) White light image of a fluorescently-labeled pancreatic adenocarcinoma tumour grown in a mouse dorsal skin window chamber; (**b**) Fluorescence image of the tumour shown in (**a**); (**c**) Tumour fluorescence image imported to red–green–blue (RGB, 256 gradations) colour space, thresholded and contoured using MATLAB software (threshold level equal to 77 out of 256); (**d**) Depth-encoded image of the corresponding 3D microvascular svOCT image co-registered with the fluorescence image contour in the en-face plane (white dashed curve, svOCT image is depth-encoded for better visual representation); (**e**) 3D tumour microvascular image generated from a 2D tumour mask projected from below the window chamber glass to the full image depth. Scale bars are 1 mm.

The VOI selection was performed in several steps as shown in Fig. 1. The tumour VOI masks are defined laterally via manual co-registration between the white-light image of the tumour (Fig. 1a) and the maximum intensity projection of an svOCT acquisition on the same day. A user-guided operation of determining the corresponding pairs of fiducials to enable automatic computation of similarity geometric transformation matrix was then implemented. This was determined to have a reproducibility of 14 μm and an accuracy of 50 μm based on testing repeated registrations of a white-light image and its corresponding svOCT acquisition. Once the similarity matrix is determined and optimized, it is applied to the fluorescence image which is intrinsically co-registered to the white light image (both taken on the same microscope) (Fig. 1b) after its binarization (Fig. 1c), which was taken immediately following the white-light image without repositioning; this finally yields the laterally co-registered tumour ROI mask (white dashed curve in Fig. 1d, where svOCT image is depth-encoded for better visual representation). Axially the tumour VOI represents the logical conjunction of the cylindrically projected tumour ROI mask and a 3D interpolation of a manually drawn tumour surface contour mask every 150 μm (every 20 B-scans) based on the raw structural OCT acquisition (prior to svOCT-processing)^39^. Finally, this tumour mask was projected from below the window chamber glass to the full image depth (~ 2.03 mm) (Fig. 1e). Note that in a typical manual metric extraction, the data post-processing is restricted to a depth of ~ 1 mm where the SNR is sufficiently high to permit relatively reliable vascular segmentation^39^. However, for the purpose of this CNN analysis the full acquired depth of approximately 2 mm was considered, so that the newly proposed methodology makes full use of acquired OCT microvascular data.

After the application of the tumour VOI, a series of denoising steps consisting of morphological and Gaussian filters were employed to reduce the background signal intensity, increase contrast, and remove some of the “salt-and-pepper” speckle noise in the 3D svOCT microvascular images^11,40^. The extent of these image pre-processing steps was fairly modest and direct, compared to the more extensive and more subjective set of operations and transformations (e.g., binarization, skeletonization, etc.) that are needed for analytical metrics derivations^9,13–16^. The resultant microvascular images were then grouped for AI analysis. As summarized in Table 1, there were 13 image volumes in the low-dose (10 Gy) category and 14 image volumes in the high-dose (30 Gy) category obtained from 10 different mice, and imaged at different time points 2–4 weeks following irradiation. Such a dataset was chosen to maintain consistency of imaged time points across all mice and because previous analyses have noted the significant discrepancy between dose cohorts at this time range^9,39^. The dataset was then repeatedly split into a training set for the CNN model to learn from, and a testing set for examining its performance. This was done five separate times so as to have five different independent training–testing set combinations; the corresponding results for these different combinations were not pooled but are reported separately.

**Table 1:** Two irradiated mice cohorts comprised of svOCT microvascular volumetric image sets obtained at time t = 2–4 weeks following irradiation.

Dose level (class)	Mouse	Time points (days)
Low dose (10 Gy)	L1	17, 24, 31
	L2	17, 24, 31
	L3	15, 22
	L4	15, 24, 29
	L5	15, 21
High dose (30 Gy)	H1	16, 25, 32
	H2	15, 22
	H3	14, 22, 29
	H4	14, 22, 29
	H5	17, 26, 32

**Figure 2:**
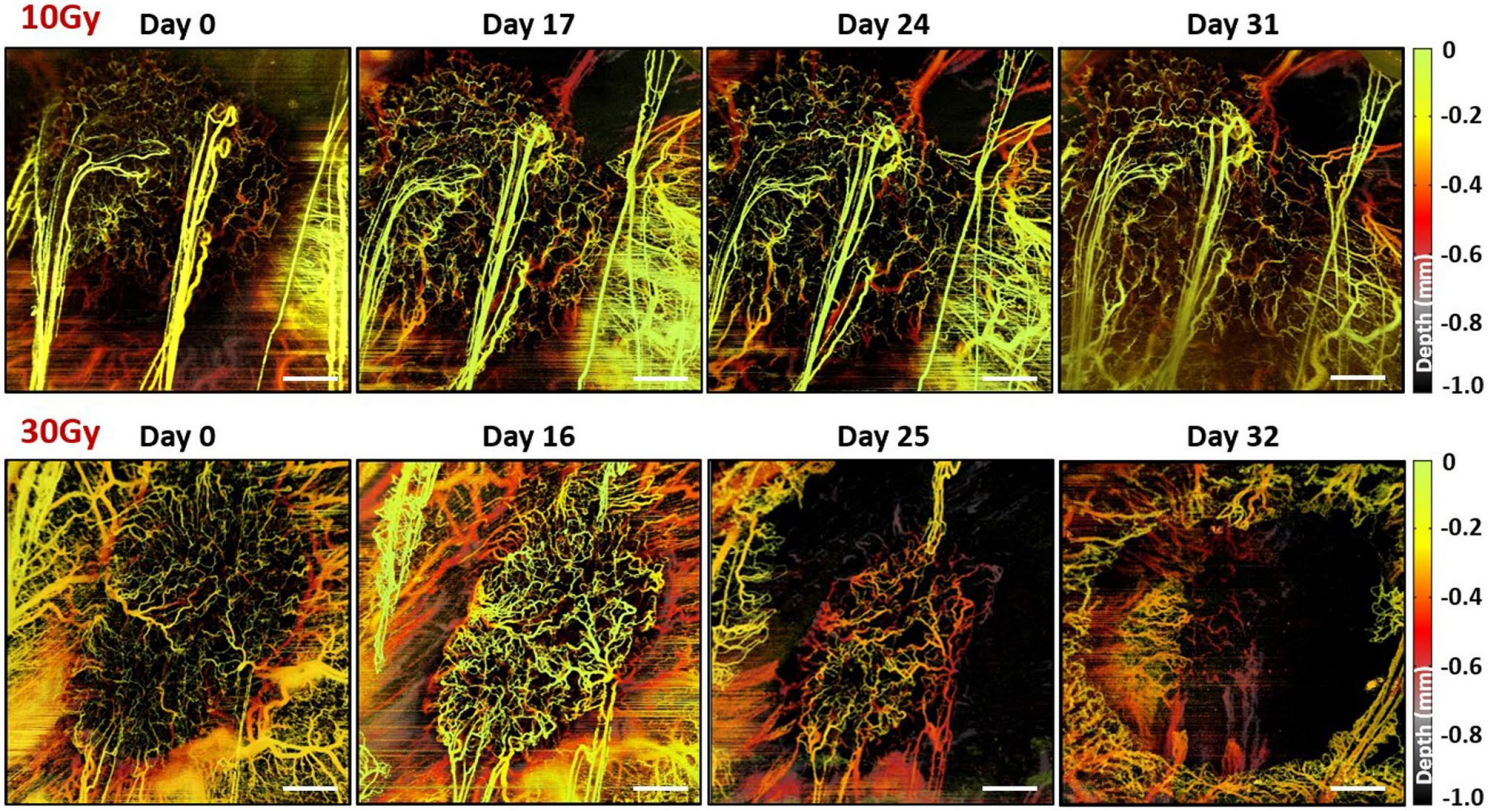
Tumour microvasculature images (before masking, depth-encoded here for better representation) used in analysis: (Top row) Days 0, 17, 24, and 31 time points of a tumour irradiated with 10 Gy; (Bottom row) Days 0, 16, 25, and 32 time points of a tumour irradiated with 30 Gy. Scale bars are 1 mm. Day 0 images for both cases are added for reference to initial tumour size, and to emphasize inter-tumour microvascular variability.

Representative examples of tumour microvascular images used in CNN analysis are shown in Fig. 2. Four angiograms of tumours irradiated with 10 Gy (top row) and 30 Gy (bottom row) illustrate the typical microvasculature response over time. From before RT (day 0) to > 4 weeks post RT microvasculature responded markedly different following (or possibly driving^9^) the changes in tumour size. Such a noticeable contrast was an encouraging starting point for development of our CNN model, with the future aim to get to more complex CNN architectures to recognize finer dose gradations even without visual differentiation.

The observed thin horizontal streaks which appear along the B-scans (orthogonal to the slow laterally galvanometer motion) are likely a result of the animal’s skin muscle spasms. These are more prominent in the thin tissue surrounding the relatively rigid and more massive tumour. They can eventually be removed via morphological opening and closing operations previously mentioned for removal of “salt & pepper” noise. We are working on a methodology to automate the associated pre-processing, including the determination of the optimal parameters for this morphological filtering step.

Each training set employed image sets from 8 mice (4 for each class) and the testing set has images from 2 mice (1 for each class). The mice in the five combinations were completely separate with no overlap between them i.e., no repeated mice combinations, and no time points from the same animal across the training and testing sets. This strict separation was important to prevent any possible overfitting/data train-test ‘contamination’, especially given the modest size of the overall data set. Indeed, as having only 27 unique volumetric image sets is insufficient for any reasonable CNN analysis, the data was scaled up using image augmentation techniques to 232 images. Specifically, the training set now had 200 image volumes (100 for each class) and the testing set had 32 (16 for each class), yielding an approximately 85:15 split ratio. The augmentation used 3D horizontal/vertical flips and rotations at various angles between − 40° and 90°, chosen arbitrarily, on each original image so as to increase the size of the dataset. Neural networks in general require larger numbers for optimum training, but the information content of the high-resolution 3D svOCT microvascular images proved sufficient for our purpose even at these modest augmented numbers.

**Figure 3:**
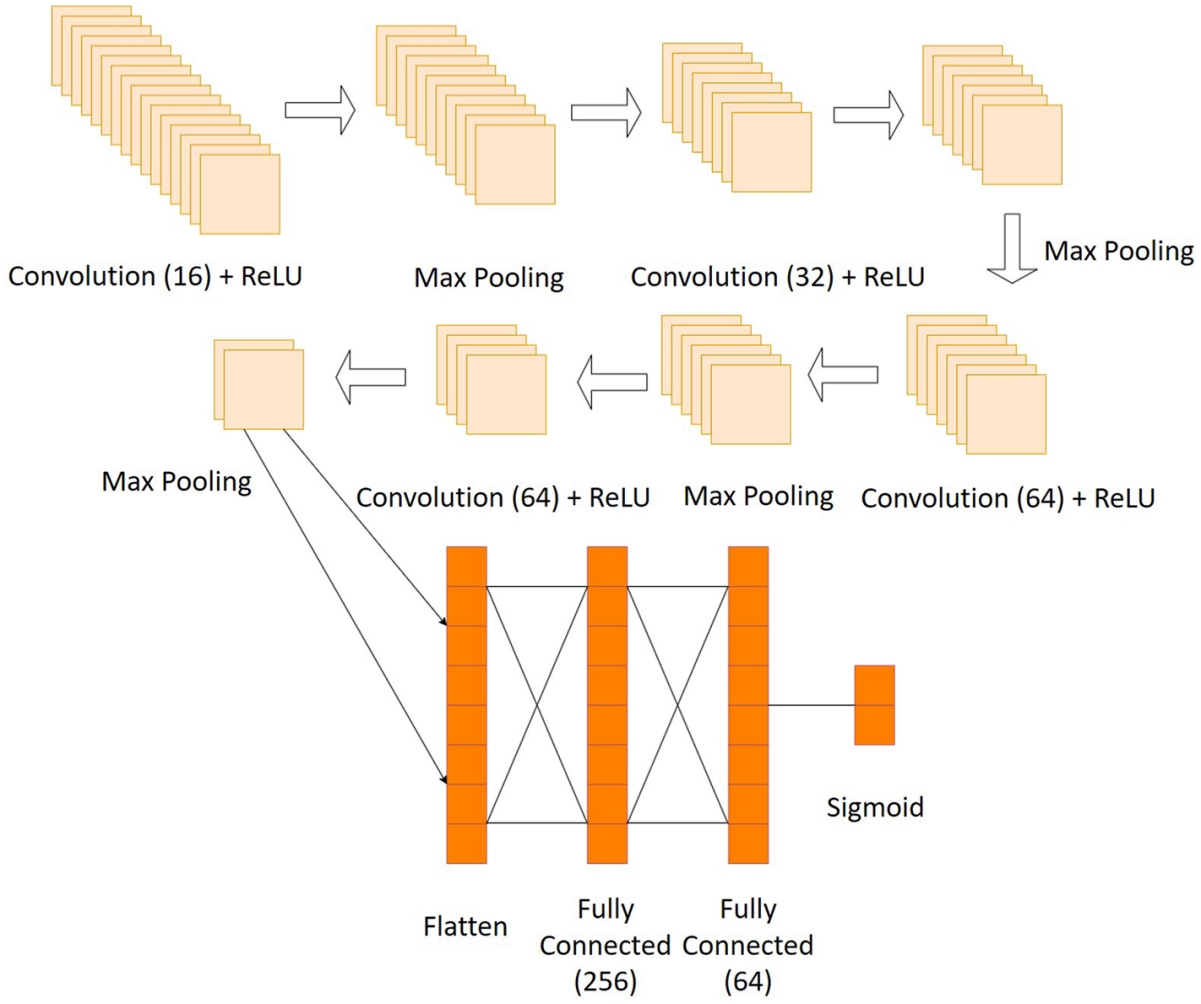
Summary of CNN model architecture for the binary problem. A 4-layer convolution network has been used with 16, 32, 64, and 64 3 × 3 × 3 sized filters each. The activation function for each convolution layer is a rectified linear unit (ReLu)^41^ along with a 2 × 2 × 2 sized max pooling (feature aggregating) layer. The output from the hidden convolution layers is flattened, after which there are two fully connected (dense) layers with 256 and 64 units (for the final layer) respectively. The final activation layer is a sigmoid function^42^ returning the 2-class membership probabilities as output. The optimizer used for the model is Adam^43^ with a step size of 0.0001, and the loss function used is binary cross-entropy^44^.

The resultant 3D images in the dataset were resized to a standard 200 (X) × 200 (Y) × 400 (Z) pixel sizes and loaded into the memory along with their corresponding labels. An input tensor was then created in the format required for the 3D CNN implementation of the form (*n* samples, length, width, depth, colour channel). Here, *n* is the number of samples, the length, width & height are dimensions of the 3D image, and colour channel (= unity) represents the single gray-scale colour of the tumour vasculature in the image volumes. The CNN architecture for the binary classification problem is displayed in Fig. 3. The CNN models were trained on a computer with a 6-core i7-9750H CPU running at 2.60 GHz with 16 GB RAM. The training time for each model averaged around 12.5 hours.

## Results and Discussion

**Figure 4:**
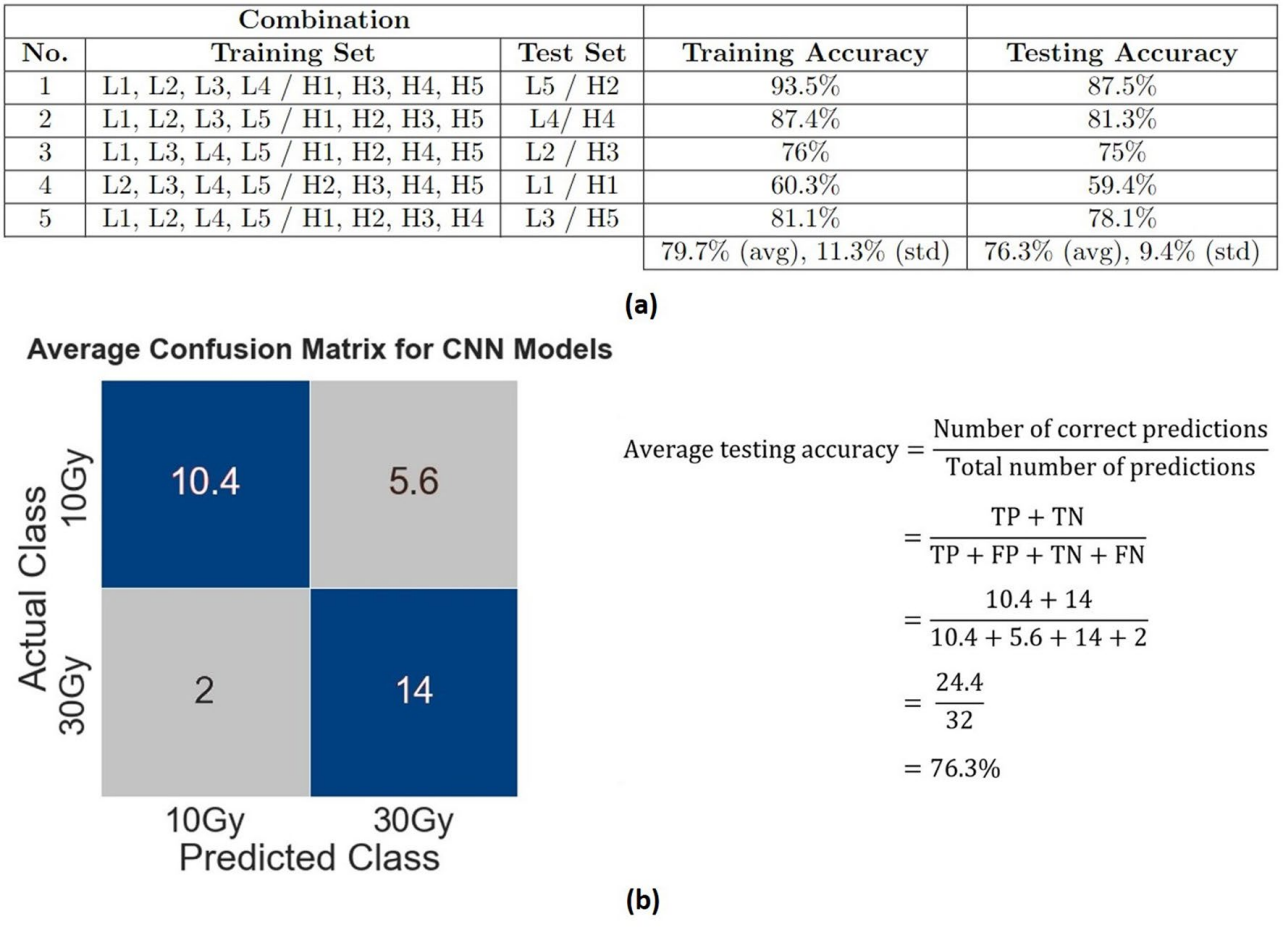
Performance summary of the models in terms of accuracy, and identification of the best model: (**a**) Training and testing accuracy of the 5 separate CNN train-test runs (avg: average, std: standard deviation); (**b**) Average confusion matrix and testing accuracy generated by the five CNN models for the binary low dose vs. high dose classes problem using 200 training image volumes and 32 testing image volumes (as evident from N = 32 summed up over all quadrants of the matrix). Blue quadrants represent the average number of correct predictions (true positives & true negatives) taken from each confusion matrix generated from each of the 5 runs; grey quadrants represent the average number of incorrect predictions (false positives & false negatives). The decimal values in the top two quadrants occur due to the sum of the predictions not being evenly divisible by five, unlike the bottom two which do yield whole numbers. *TP:* true positives, *FP:* false positives, *TN:* true negatives, *FN:* false negatives.

In order to assess the predictive performance of the model for the binary low dose (10 Gy) *versus* high dose (30 Gy) classification problem, we examine the accuracy scores given by each of the five independently analyzed train-test CNN models. Figure 4a tabulates the individual testing accuracy scores for the 5 separate examined data set combinations, indicating an accuracy range of 59–88%. The large spread is likely related to the low number of the unique imaged data sets, despite our efforts at data augmentation. We also look at the resultant confusion matrix which summarizes the predictions on testing data for both classes in the form of the number of true positives (low doses being correctly predicted as low dose), true negatives (high dose being correctly predicted as high dose), false positives (low dose being incorrectly predicted as high dose), and false negatives (high dose being incorrectly predicted as low dose). Figure 4b displays the average of the five confusion matrices generated by the five independently performed train-test CNN models to give an idea of the average performance across the five models.

The results are encouraging, with an average testing accuracy score of 76.3%. Despite the spread in the results, the overall reasonably high resultant accuracy values suggest that this proof-of-concept AI pipeline can distinguish between low and high doses levels well based on 3D microvascular svOCT images, commensurate to the performance of comparable classifiers in the context of published OCT reports^19,20,27–29,32–37^.

**Figure 5:**
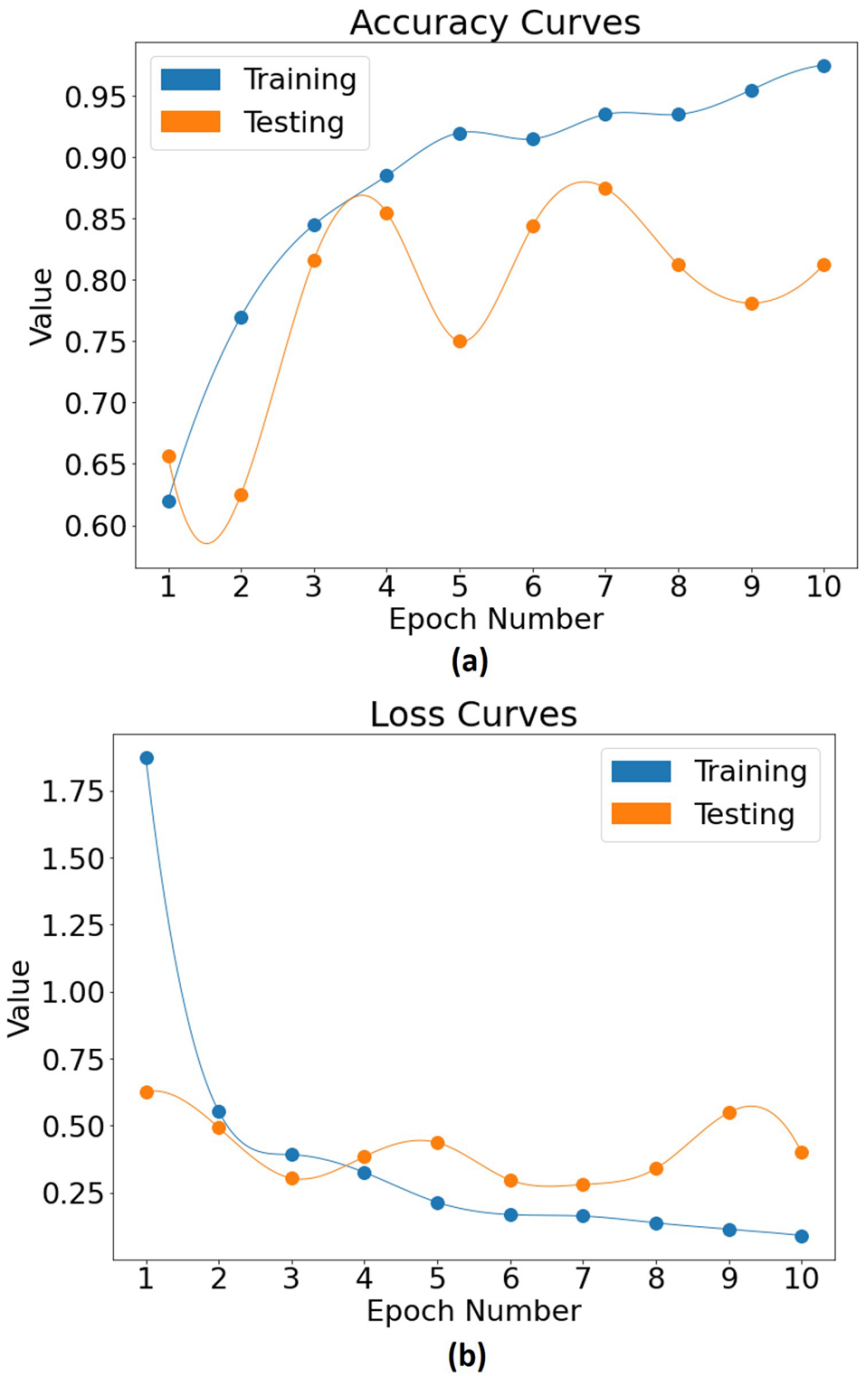
Performance curves of the CNN model for run #1 in terms of: (**a**) accuracy score, ranging between 0 and 1; (**b**) loss function values, ranging between 0 and the highest observable error (~ 2) for this specific run, both plotted against the number of epochs for the training (blue) and testing (orange) sets. Results are represented by circular symbols and the cubic spline curves are guides for the eye. The point of divergence between the training and testing curve in both (**a**) and (**b**) begins at the 7th epoch, making it the point of the optimal trained state of the CNN model, with a testing accuracy of 87.5% and training accuracy 93.5%. For details, see text.

For additional insight, we examine the CNN performance of a particular train-test dataset, for example run #1 (top entry in Fig. 4a). Figure 5a shows the evolution of the training and testing accuracy as a function of epoch (train cycle) number. Training accuracy here refers to the predictive ability of the model on the training set i.e., the known images from which the model learns from. Although perhaps inessential as a metric by itself, looking at the training accuracy is a means to monitor the learning process and adjust the various hyperparameters of the CNN model. The more important testing accuracy is seen to be fluctuating over the course of 10 epochs, likely due to the small size of the testing set making the stochastic gradient descent process wander around the local minimum.

Figure 5b shows the loss function evolution for both training and testing sets, which indicates the prediction error of the CNN model at each epoch. These loss values are used by the CNN model at every epoch to update its weights so as to minimize the error and perform better on the next run. As the testing accuracy is fluctuating, it is important to look at the corresponding loss value in parallel since a high test accuracy might potentially have a large prediction error, meaning the predictions are made with a low “confidence”. As an example, for the data shown here, a testing accuracy of 85.5% is observed at the 4th epoch but with a higher loss value as compared to later epochs.

In order to assess when the model has been optimally trained, we look at both the accuracy and loss curves in Fig. 5a,b. In both cases, after the 7th epoch, the training performance keeps getting better whereas the predictive capability of the model goes down. This divergence indicates that overfitting has begun i.e., the model starts memorizing the input samples and not generalizing on the testing data well enough. This is why the 7th epoch is considered as the point where the model is optimally trained (testing accuracy 87.5%, training accuracy 93.5%). Although the above discussion pertains to run #1, similar considerations apply to the other 4 cases as well. However, each of the 5 runs in this study appear to reach their optimal performance at a different epoch number (specifically epoch # = 6 for run #2, 5 for run #3, 2 for run #4, and 7 for run #5), and their accuracy/loss curves have somewhat different shapes and points of divergence. The large spread in the testing accuracy results (tabulated in Fig. 4a) is consistent with this behaviour. Overall, this suggests that the composition of the 3D microvascular svOCT images in the training and testing sets influences the way the CNN model learns to distinguish between low dose versus high dose, and the time taken to do so. The possible reasons behind this might have to do with the differences in the amount of vessels present in the VOIs, and the variability of the tumour vasculature across the training and testing sets. The addition of more images in the future may yield a more uniform training and testing behaviour.

To put this work in a larger context, we note that the analytical metrics calculated by us and others from the svOCT microvascular images such as vascular volume density, fractal dimension, average segment length, tortuosity, and so forth can quantify microvascular network and may have a role in (radio)therapy monitoring scenarios^9,11,12^. These however are not easy to derive accurately, requiring a significant amount of image processing. Further, their selection is expertise-dependent and thus somewhat subjective. In contrast, deep learning methods explored in this study are considerably less reliant on image processing and on researcher expertise, while still categorizing the irradiated vasculature into low- and high-dose classes with reasonable success. These AI methods can also potentially make use of the entire 3D heterogeneity of the vascular patterns and its changes in response to therapy, something that the analytical single-number-summary metrics do not do well^9^. This purported sensitivity to the biomedically-important tumour spatial heterogeneity is yet to be proven rigorously and will be pursued in our future studies.

It is also important to note that the relatively simple problem of low- versus high-dose categorization is probably solvable without the advanced AI algorithms presented here. For instance, quantification of various analytical biomarkers discussed previously, or the use of ‘avascular holes’ metric recently reported by Allam et al.^39^ would likely prove sufficient in this case. Yet the approach used in this proof-of-principle study does indicate what may work for the more challenging and more interesting problems, and the structure of the complete deep learning pipeline developed here will enable direct adaptation to newer and more challenging investigations. For example, the data augmentation and epoch number optimizations issues from the current work suggest that the incorporation of additional svOCT volumes at various time points is necessary to have an increased overall dataset size for more consistent results. We are therefore currently adapting this convolution neural network platform to look at finer dose levels, to examine the temporal evolutions of the dose–response relationship, and to predict treatment outcomes based on early microvascular responses.

The promise of AI-accelerated, more in-depth, and objective data analysis may open the doors to further interesting experiments. One direction can be the investigation of more realistic tissue response models (beyond the partially invasive window chamber preparation) such as subcutaneous tumour inoculations employed in conjunction with optical clearing methods^45,46^ as recently proposed by Kistenev et al.^47^. This could lead to more reliable deep learning algorithms^48^, yielding a robust and detailed picture of biological response to ionizing radiation including SBRT^3,49^.

## Conclusion

This study explored and demonstrated the potential benefits of utilizing artificial intelligence in the context of svOCT volumetric imaging of tumour vasculature in response to radiation therapy. The goal was to classify the svOCT images of tumour vasculature into their two SBRT-relevant clinical radiation dose levels (10 and 30 Gy, respectively). A convolution neural network was employed, yielding an average testing accuracy of 76.3% with five separate train-test 3D image sets. Although the results are promising, it is important to note that their statistical reliability can vary depending on the type of image augmentations employed and corresponding images generated for the dataset. Testing images of types other than the ones used for training the models or coming from a different distribution may result in a different accuracy. Moving ahead from this pilot study, the aim should be to expand the feature space used by these models as much as possible to have consistent results with the testing data. The advantages of the AI approach over analytical metrics quantification and analysis include simpler image processing, less reliance on researcher expertise and thus more objective outcomes, and potential ability to better capture tumour spatial heterogeneity. Issues with modest dataset sizes, image augmentation mitigations, and epoch training cycle optimizations were identified and discussed. Looking ahead, with higher processing power and availability of a GPU for training, a variety of additional benchmarks can test the performance of more complex CNN architectures and speed up training time to tackle advanced problems involving more dose gradations, longitudinal response monitoring, and clinical outcome predictions. Adaption of the developed AI platform towards these advanced ‘shedding light on radiotherapy’ studies is currently ongoing in our laboratory.

## Data Availability

The datasets generated and analyzed during the current study are not publicly available due to its sensitive nature but may be obtained from the corresponding author on reasonable request.
